# The Unsolved Puzzle of c-Rel in B Cell Lymphoma

**DOI:** 10.3390/cancers11070941

**Published:** 2019-07-04

**Authors:** Maike Kober-Hasslacher, Marc Schmidt-Supprian

**Affiliations:** 1 Institute of Experimental Hematology, School of Medicine, Technical University Munich, Ismaninger Straße 22, 81675 Munich, Germany; 2 German Cancer Consortium (DKTK) and German Cancer Research Center (DKFZ), 69120 Heidelberg, Germany

**Keywords:** c-Rel, NF-κB, B cells, *REL* gene locus amplification, lymphoma, FL, DLBCL, PMBCL, cHL

## Abstract

Aberrant constitutive activation of Rel/NF-κB transcription factors is a hallmark of numerous cancers. Of the five Rel family members, c-Rel has the strongest direct links to tumorigenesis. c-Rel is the only member that can malignantly transform lymphoid cells in vitro. Furthermore, c-Rel is implicated in human B cell lymphoma through the frequent occurrence of *REL* gene locus gains and amplifications. In normal physiology, high c-Rel expression predominates in the hematopoietic lineage and a diverse range of stimuli can trigger enhanced expression and activation of c-Rel. Both expression and activation of c-Rel are tightly regulated on multiple levels, indicating the necessity to keep its functions under control. In this review we meta-analyze and integrate studies reporting gene locus aberrations to provide an overview on the frequency of *REL* gains in human B cell lymphoma subtypes, namely follicular lymphoma, diffuse large B cell lymphoma, primary mediastinal B cell lymphoma, and classical Hodgkin lymphoma. We also summarize current knowledge on c-Rel expression and protein localization in these human B cell lymphomas and discuss the co-amplification of *BCL11A* with *REL*. In addition, we highlight and illustrate key pathways of c-Rel activation and regulation with a specific focus on B cell biology.

## 1. Introduction: c-Rel Is the NF-κB Family Transcription Factor with the Strongest Link to Human Lymphoma

The transcription factor c-Rel is one of five members of the nuclear factor κ-light-chain-enhancer of activated B cells (NF-κB) family of transcription factors. In contrast to other ubiquitously expressed Rel/NF-κB family members [1], high c-Rel expression has been predominantly detected in the hematopoietic lineage, under healthy conditions [2]. The particular importance of c-Rel function in the immune system, in general, and in B cells, in particular, was revealed through the analyses of conventional and conditional c-Rel knockout mice [3,4,5,6]. During steady state conditions, dimers of NF-κB proteins are kept inactive sequestered in the cytoplasm through interaction with inhibitor of κB (IκB) proteins. Various upstream stimuli mark these IκB proteins for proteasomal degradation allowing homo- or heterodimeric NF-κB dimers, including c-Rel complexes, to translocate to the nucleus to reprogram gene expression [7,8]. The c-Rel/NF-κB target gene space is characterized by redundancy through substantial overlap and compensation between the NF-κB subunits [1]. Key c-Rel/NF-κB targets include genes encoding survival factors, regulators of cell cycle, and proliferation, as well as mediators of immune cell signaling [9]. Given these groups of target genes, it is not surprising that aberrant constitutive NF-κB activation is a hallmark of numerous cancers, including lymphoid tumors [10,11,12]. Intriguingly, to date, c-Rel is the only member of the NF-κB family for which direct transforming activity has been shown: Retroviral expression of both human and mouse c-Rel led to malignant transformation of chicken spleen cells in vitro [13].

In this review, we discuss literature that lays the foundation for the current picture of c-Rel’s role in human B cell lymphomas. We begin with an introduction of c-Rel signaling by highlighting aspects of c-Rel activation and regulation, particularly in B cells. We then focus on the frequent occurrence of *REL* gene locus gains and amplifications in human B cell lymphoma and provide an overview of reported gene locus aberrations in relevant human lymphoma subtypes. Furthermore, we summarize publications analyzing c-Rel expression and protein localization in these human B cell lymphomas and discuss the co-amplification of *BCL11A* with *REL*.

## 2. Control of c-Rel Expression, Abundance, and Activation in B Cells

**Figure 1:**
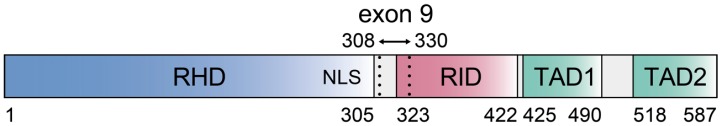
Human c-Rel protein domains—schematic illustration. Amino acid start and end points of represented protein domains are indicated by numbers below the scheme. The position of the amino acid sequence encoded by exon 9 (aa 308–330) is highlighted by dotted lines. RHD, Rel homology domain; RID, Rel inhibitory domain; TAD, transactivation domain; NLS, nuclear localization signal. This figure is based on [9,15]. Other references assign the RHD to aa 8–290 [21] or aa 8–297 (UniProt database, UniProtKB, Q04864 REL (human), www.uniprot.org).

The human *REL* gene locus on chromosome 2 encodes the c-Rel protein with a length of 587 amino acids and an approximate molecular weight of 65 kDa [14,15] (Figure 1). The first 300 amino acids at the c-Rel amino terminus constitute the highly conserved Rel homology domain (RHD), which is shared with other NF-κB family members. The RHD is involved in DNA-binding, dimerization, inhibitor interaction, and nuclear localization [7]. At its carboxy terminus, c-Rel contains a transactivation domain (TAD), which harbors two subdomains referred to as TAD1 and TAD2 that map to amino acids 425–490 and 518–587, respectively [9,16,17]. The protein sequence upstream of the TAD at amino acids 323–422 was defined as the Rel inhibitory domain (RID) as mutants lacking this region show enhanced transactivation and DNA-binding in vitro [15]. c-Rel carries a nuclear localization signal (NLS) but no nuclear export signal (NES) [18,19]. Remarkably, two alternative versions of the *REL* transcript were identified in human B cell lymphoma: First, a *REL* transcript containing an exonized Alu element between exon 8 and 9 that could encode a protein of 619 amino acids [20], second, a lymphoma-specific splice variant of human c-Rel lacking the entire exon 9 (amino acids 308–330) with a higher in vitro transactivation activity [15].

In the mouse, under normal physiological conditions, high expression of c-Rel is predominant in the hematopoietic system [2]. c-Rel expression is regulated by transcription factors of the PU.1/SpiB family [22,23] as well as through NF-κB transcription factors, including c-Rel itself [24]. The autoregulation of c-Rel and other NF-κB family members, including a database mapping of the binding sites for RelA, RelB, and c-Rel itself within the c-Rel promoter has been recently reviewed [25].

c-Rel is highly abundant in resting splenic B cells and its expression is dramatically upregulated upon activation. This is in line with a predominant NF-κB dimer composition of c-Rel and p50 in mature B cells [26]. Several lines of evidence point to an important role for PI3K signaling in maintaining c-Rel levels in B cells [27,28,29]. It was estimated that 20% of mature resting B cells contain nuclear c-Rel [26,30], pointing to a role downstream of receptor signals regulating normal B cell homeostasis. Upon appropriate activation in the context of immune reactions, B cells differentiate into germinal center (GC) B cells and antibody-producing plasma cells. Gene expression profiling revealed decreased c-Rel mRNA in mouse plasmablasts and plasma cells compared to mature B cells [31]. Downregulation of c-Rel mRNA [32] and c-Rel protein expression [33] was also found in human plasmablasts/plasma cells. This is in accordance with gene array as well as RNAseq data assembled by the ImmGen consortium, which show a continuous decrease of c-Rel mRNA from mature resting B cells via GC B cells to plasma cells [34]. Nevertheless, c-Rel plays a critical role in terminal B cell differentiation, as its specific ablation in GC B cells by conditional gene targeting leads to loss of GC B cells and strongly impaired antibody responses [6]. A recent study suggested that c-Rel expression in plasmablasts is downregulated through Blimp-1-mediated repression by direct binding to the c-Rel promoter, which facilitates plasma cell differentiation [35]. In human, high c-Rel expression was detected in GC B cells. c-Rel predominantly localizes in the cytoplasm with rare instances of nuclear localization [36]. GC B cells with nuclear c-Rel are located in the light zone of the GC [37], where GC B cells receive NF-κB-activating signals from T cells. These studies show that c-Rel expression is tightly and dynamically regulated at the transcriptional level during B cell development and functional differentiation, reflecting its critical roles in B cell functions. While we focus on aspects of c-Rel in terminal B cell differentiation in this review, previous excellent reviews cover the complexity and interplay of the NF-κB family members in B cell development and beyond—including studies on the basis of mouse models [38,39,40].

**Figure 2:**
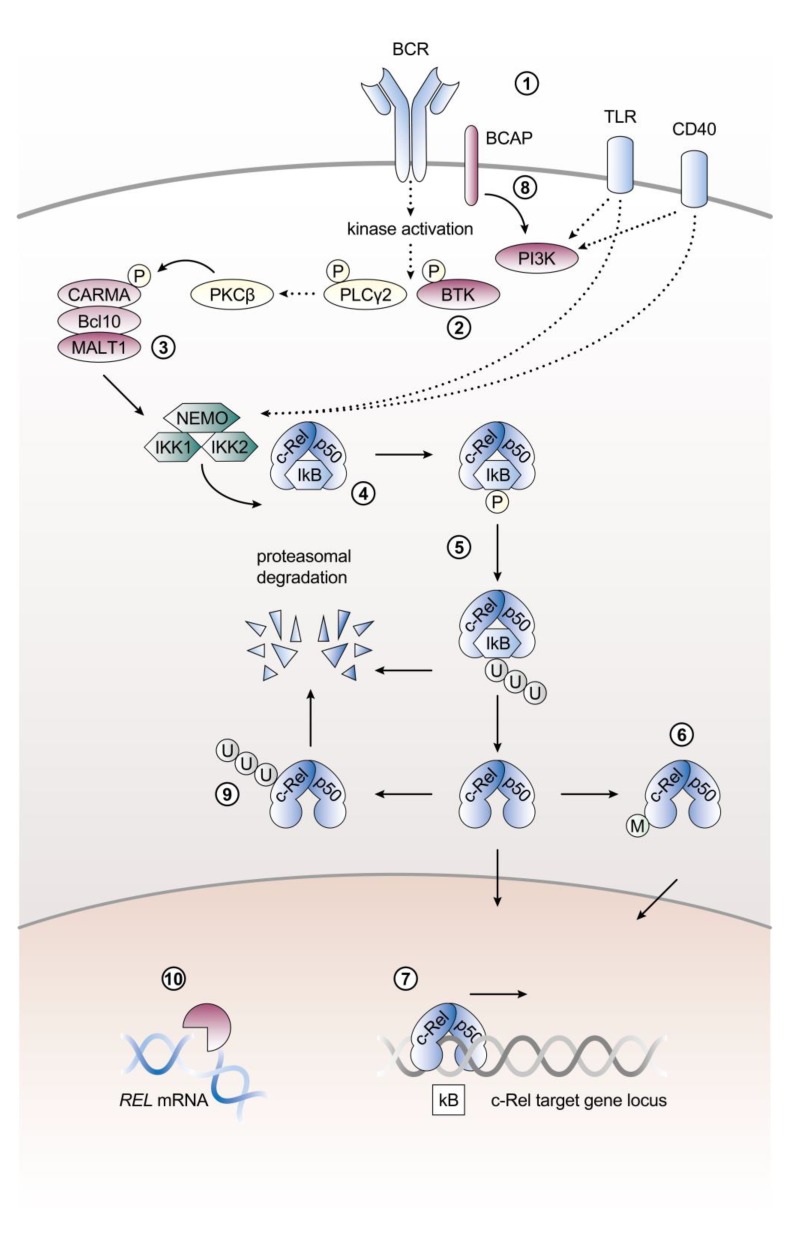
Activation of c-Rel signaling by the canonical NF-κB pathway in B cells. Major signaling and regulatory components described in the main text are illustrated. (**1**) Cardinal triggers of c-Rel signaling include B cell receptor (BCR), toll-like receptor (TLR) or CD40 stimulation. (**2**) In Btk-deficient B cells c-Rel DNA-binding activity is strongly reduced following stimulation. (**3**) The paracaspase mucosa-associated lymphoid tissue protein 1 (MALT1) which is part of the CBM complex is specifically required for BCR signal-induced c-Rel nuclear translocation. (**4**) In mature B cells the predominant NF-κB dimers are formed by c-Rel and p50. (**5**) c-Rel is sequestered in the cytoplasm by interaction with the inhibitory proteins IκBα, IκBβ and IκBε. Various upstream stimuli of canonical NF-κB signaling can target these IκB proteins for proteasomal degradation. (**6**) c-Rel is modified on a post-translational level. These post-translational modifications (M) can influence c-Rel transactivation and transforming activity. (**7**) Once released from the inhibitory IκB proteins, c-Rel translocates into the nucleus and binds to κB target sites to exert its function as a transcription factor. (**8**) PI3K signaling contributes to maintaining c-Rel levels in B cells. (**9**) Regulation of c-Rel is mediated by ubiquitination and subsequent proteasomal degradation. (**10**) *REL* mRNA levels are controlled on a post-transcriptional level. References as well as further details and abbreviations are provided in the main text. In addition to references cited in the main text, the content of this figure is based on Okkenhaug and Vanhaesebroeck, 2003 [65], Siebenlist et al. 2005 [66], and Murphy et al. 2007 [67]. P, phosphorylation; U, ubiquitination; M, post-translational modification.

The activation of c-Rel is initiated by diverse stimuli and tightly regulated through multiple mechanisms. Mechanisms regulating c-Rel activity include enhanced expression, control of *REL* mRNA stability and translation on the post-transcriptional level, receptor-induced signaling cascades, release of sequestration by IκB proteins, nuclear shuttling as well as post-translational modifications (Figure 2).

In B cells, c-Rel is activated by cardinal triggers of canonical NF-κB signaling, including antigen recognition by the B cell receptor (BCR), receipt of T cell help through CD40 as well as sensing microbial products via toll-like receptors (TLRs), namely TLR4 and TLR9 [9,40]. BCR signal-induced c-Rel nuclear translocation specifically requires a signal transduction complex composed of the paracaspase mucosa-associated lymphoid tissue protein 1 (MALT1), caspase activation and recruitment domain (CARD)-containing membrane-associated guanylate kinase 1 (CARMA1) and Bcl-10, also known as the CBM complex [30,41]. Upon BCR stimulation MALT1 is required to dissociate c-Rel, but not RelA, from IκBα and IκBβ. This MALT1-dependence is specific for signals downstream of the BCR as LPS/TLR4-initiated c-Rel nuclear translocation is not impaired in MALT1-deficient B cells [30]. c-Rel activation requires MALT1′s function as a protease, as MI-2, an irreversible direct enzymatic inhibitor of MALT1, was shown to inhibit c-Rel nuclear translocation in human lymphoma cell lines in vitro and in a xenograft mouse model [42]. Moreover, Bruton’s tyrosine kinase (Btk) plays a role in c-Rel activation as c-Rel DNA-binding is strongly reduced in Btk-deficient B cells upon BCR engagement or treatment with B cell activating factor (BAFF), despite unchanged c-Rel protein levels [43].

In addition to its role in acute responses, c-Rel plays a critical role in sustaining longer-lasting NF-κB responses in B cells. Damdinsuren et al. found that continuous BCR-engagement leads to de novo c-Rel induction and nuclear c-Rel accumulation at 6 to 24 h, correlating with enhanced expression of pro-survival genes, while nuclear quantities of RelA are negligible during this time period [44].

In contrast to RelA, c-Rel does not contain a nuclear export signal (NES). As a consequence, RelA-containing dimers are more efficiently shuttled out of the nucleus compared to c-Rel complexes in vitro [18]. As initial NF-κB activation potently induces de novo c-Rel expression, nuclear-export resistant c-Rel complexes dominate the prolonged NF-κB response [1,44].

IκB proteins represent a fundamental layer of restraining NF-κB activation. It was proposed that in pre-B cell lines c-Rel is mainly associated with IκBβ, whereas c-Rel is associated with both IκBα and IκBβ in mature B cells. This implicates that differential c-Rel:IκB associations could underlie the selective activation of NF-κB subunits at individual stages of B cell development [18,45]. The significance of IκBα localization and degradation in the regulation of c-Rel activity is demonstrated in a mouse model expressing a mutant IκBα lacking a functional IκBα NES. These mice show severe defects in the B cell lineage that in part phenocopy c-Rel knockout mice [26]. A third IκB protein, IκBε, inhibits c-Rel in lymphocytes. B cells from IκBε-deficient mice are characterized by an increase in basal nuclear c-Rel and enhanced nuclear DNA-binding activity of c-Rel-containing complexes upon anti-IgM or LPS treatment [46,47].

The amount of c-Rel protein present in cells is regulated at the post-transcriptional level by RNA-binding proteins controlling *REL* mRNA stability and translation. In T cells *Rel* mRNA is a substrate for the ribonuclease Regnase-1 that cleaves its target mRNAs mostly in the 3′UTRs [48]. In addition, *Rel* mRNA is also a target of the RNA-binding Roquin1 and Roquin2 paralogs that induce mRNA decay by binding to 3′UTRs of target mRNAs [49]. Both Regnase-1 [48] and Roquin proteins [50] are also expressed in B cells. However, the precise in vivo impact of these mRNA-destabilizing pathways on c-Rel protein levels and function in B cell physiology and pathology remains to be delineated.

c-Rel can be modified by various post-translational modifications [9,51]. Several publications provide evidence for protein phosphorylation of c-Rel within its carboxy-terminal TAD. In vitro studies suggest that NF-κB inducing kinase (NIK) [52] or TNFR-associated factor family member-associated NF-κB activator (TANK)-binding kinase 1 (TBK1) and IKKε [53] can phosphorylate c-Rel, which is associated with enhanced transactivation or nuclear accumulation, respectively. Interestingly, NIK mutant B cells contain strongly reduced levels of c-Rel [54]. c-Rel phosphorylation is not only implicated in transactivation by further studies [16,55,56] but also in strengthening its transforming activity of lymphoid chicken cells in vitro [57,58]. In addition, the redox status of a cysteine residue in the RHD correlates with c-Rel phosphorylation and DNA-binding abilities [59]. Furthermore, mutations in the RHD within a putative protein kinase A (PKA) recognition site were proposed to render c-Rel temperature-sensitive [60]. The ubiquitin proteasome system as a means to regulate c-Rel turnover was first reported in in vitro studies that suggested that a carboxy-terminal part of c-Rel is important in promoting degradation [61]. A more recent study in mice established the E3 ligase Peli1 as an essential negative regulator of c-Rel activity in T cells. Peli1 mediates lysine-48(K48)-linked ubiquitination of c-Rel, thereby tagging it for subsequent proteasomal degradation [62]. c-Rel can also be modified by glycosylation, specifically through the addition of O-linked β-N-acetyl-glucosamine (O-GlcNAcylation) to Serine 350 in vitro, which was suggested to enhance the transcription of c-Rel target genes [63].

Finally, the peptidyl-prolyl cis/trans isomerase, NIMA interacting (Pin1) catalyzes isomerization of proline amide bonds. Pin1 was shown to associate with c-Rel and influence c-Rel nuclear translocation in human B cell lymphoma cell lines as pharmacologic inhibition or knockdown of Pin1 decreased c-Rel nuclear translocation [64].

## 3. Aspects of B Cell Lymphomagenesis

Intriguingly, approximately 95% of human lymphomas arise from B cells. The majority of human B cell lymphomas, in turn, contain somatically mutated immunoglobulin (Ig) genes, which provides strong evidence for a germinal center (GC) or post-GC origin [68]. When B cells encounter an antigen that engages their BCR while they receive co-stimulatory signals via cytokines and cell-cell contacts derived from cognate T helper cells, they initiate the germinal center reaction. During this reaction B cells vigorously proliferate and modify their B cell receptor genes through somatic hypermutation and class switch recombination. Both these physiological immune mechanisms require DNA breaks in GC B cells and an elevated tolerance for DNA damage is critical for progression through these processes. As a consequence, GC B cells are more prone to genetic instability, including translocations, deletions, mutations, and amplifications [12,68,69]. Indeed, chromosomal translocations involving Ig genes are a common feature of human B cell lymphomas [68], often resulting in the overexpression of oncogenes through strong Ig promoter and enhancer sequences. Furthermore, somatic hypermutation targets many genes in addition to Ig loci, both physiological targets such as the central GC transcriptional modulator Bcl6 as well as aberrant targets including various proto-oncogenes [70,71,72]. Interestingly, B cells harboring translocations involving Ig genes are present in healthy mice and human at very low frequency, demonstrating that additional alterations are required for full transformation [73].

The genetic aberrations that promote lymphomagenesis, also referred to as “drivers”, typically provide B cells with enhanced proliferative and survival capacities or disrupt normal cellular differentiation processes that often result in blocked or impaired differentiation [73,74]. Beyond these intrinsic determinants, the lymphoma microenvironment is of critical importance for tumorigenesis, tumor maintenance, and evolution [68]. Lymphomas corrupt their surrounding cells to change gene expression in order to support the malignantly transformed cells, and they need to escape or break immune surveillance exerted by T cells and other immune cells [75,76].

## 4. Genetic Aberrations Involving the *REL* Gene Locus in B Cell Lymphoma

Human B cell lymphomas frequently contain gains or amplifications of the *REL* gene locus on chromosome 2, within band p16.1 (2p16.1, according to genomic location of REL ENSG00000162924 accessible on www.ensembl.org (human GRCh38.p5)). As described in detail below and summarized in Table 1; Table 2, *REL* gains/amplifications predominantly occur in classical Hodgkin lymphoma (cHL) and in B cell non-Hodgkin lymphomas (B-NHL), particularly in diffuse large B cell lymphoma (DLBCL) and in primary mediastinal B cell lymphoma (PMBCL) as well as in transformed follicular lymphoma (tFL). Many reports distinguish amplifications, referring to high copy number increases from gains that may also include moderate genomic overrepresentations. Most of the surveyed studies define an amplification as a gene copy number ≥4. However, much larger copy number increases are possible, indeed a DLBCL case was found to contain more than 100 copies of the *REL* locus [77]. While Table 1 of our analysis for this literature review summarizes frequencies of *REL* gene locus copy number changes including both gains and amplifications, Table 2 features only the studies reporting amplifications with a gene copy number ≥4.

Chromosomal gains including the *REL* locus were also found in three additional types of B cell lymphoma, namely nodular lymphocyte predominant Hodgkin lymphoma (NLPHL), T cell/histiocyte-rich large B cell lymphoma (THRLBCL) [78] and B cell chronic lymphocytic leukemia (CLL) [79]. Interestingly, gains of 2p16.1 were also observed in peripheral T cell lymphoma, not otherwise specified (PTCL NOS) [80] and enhanced c-Rel expression was associated with poor response to therapy in adult T-cell leukemia/lymphoma (ATLL) [81]. Other genetic aberrations involving the *REL* gene locus include *REL*-positive double minute chromosomes in FL [82] and *REL* translocations [83,84]. Finally, an association of cHL with a single nucleotide polymorphism near the *REL* gene locus was reported [85,86], linking cHL with a *REL* germline variant that potentially affects transcription.

Besides these hard-wired genetic alterations involving the *REL* gene locus, c-Rel protein expression in lymphoma can also be altered through aberrant splicing. These protein alterations influence c-Rel’s in vitro transactivation and transforming abilities. A c-Rel splice variant devoid of exon 9 (c-RelΔEx9) was detected in primary samples of DLBCL patients and lymphoma cell lines but not in normal lymphoid tissue [15]. Remarkably, this c-RelΔEx9 splice variant has higher activity in in vitro transactivation assays than full-length c-Rel. Furthermore, point mutations affecting serine residues in the c-Rel TAD1, and thereby precluding phosphorylation, enhance the transforming potential of c-Rel. Conversely, serine mutations resulting in phosphomimetic residues have a positive effect on c-Rel’s transactivation activities [57]. Interestingly, the identical point mutation resulting in a change of serine 525 to proline in the c-Rel TAD2, shown to strengthen c-Rel’s transformation potential in vitro, was detected in one patient with FL and one patient with PMBCL [58]. Finally, loss of either of the two c-Rel TADs or the substitution of acidic residues with alanines within TAD2 decrease c-Rel’s transactivation capabilities while increasing its transforming potency [17,87].

### 4.1. REL Gains in Diffuse Large B Cell Lymphoma

DLBCL is the most frequent form of B-NHL representing roughly 40% of all B-NHL diagnoses [12]. DLBCL is considered an aggressive lymphoma of rather broad heterogeneity, and despite initial therapy response in most patients, durable remission is only rarely accomplished [68,88]. The cell of origin in DLBCL is thought to be a GC- or post-GC-derived B cell [68].

Two early studies, analyzing apparently largely overlapping cohorts of 111 [89] or 96 [90] DLBCL cases, identified *REL* copy number amplifications of four or more copies in 23% of the patients. Alizadeh et al. defined three DLBCL subgroups classified according to distinct gene expression profiles [88]. Since then, additional improved DLBCL sub-classifications were proposed, including the recently defined DLBCL clusters based on comprehensive molecular analysis [91,92,93]. For the purpose of this meta-analysis of papers employing the older classification, we will use the following nomenclature and try to put the terms as well as their molecular and clinical associations into the historical context. The germinal center B cell-like (GCB) subgroup expresses a gene signature associated with GC B cells, whereas the gene profile of the activated B cell-like (ABC) cluster resembles in vitro activated peripheral blood cells [88]. ABC-DLBCL display elevated expression of NF-κB target genes, whereas in germinal center B cells and GCB-DLBCL NF-κB target genes are typically underrepresented. A third subgroup termed type 3 DLBCL identified in a large cohort of 240 patients expresses neither genes of the ABC nor the GCB profiling set at high levels [94]. In these studies, the subgroups were related to clinical parameters and the authors detected a superior clinical outcome for the GCB-DLBCL subgroup [88,94]. This was, however, not confirmed in reports of 58 [95] or another 46 [77] DLBCL samples. Nevertheless, a significantly better survival rate for GCB-DLBCL patients was again determined for two of the data sets [94,95] by Wright et al. using a new subgroup prediction algorithm designed to be independent of the microarray platform that differed in the two original publications [96]. In agreement with this, a correlation of the GCB-DLBCL phenotype with better overall survival was found in an independent cohort [97]. Monti et al. suggested a DLBCL classification that focuses on cell-intrinsic signaling pathways and response to the microenvironment rather than the cell of origin. When clustering their data according to Wright et al., these authors also found extended survival rates for the GCB-DLBCL subgroup [98].

Rosenwald et al. detected *REL* amplifications exclusively within the GCB-DLBCL subgroup at a frequency of 15% (17/115) using quantitative polymerase chain reaction [94]. On the contrary, applying comparative genomic hybridization (CGH) to the same patient cohort, ranks gain or amplification of 2p14-p16 as the second most frequent gain of genetic material observed in DLBCL, which is distributed equally between GCB-DLBCL (17%, 15/87) and ABC-DLBCL (15%, 12/77). Within the gains, amplifications are more frequent in GCB- (8/14) than in ABC-DLBCL (2/11) [99]. Evaluating partially the same cohort using array-based CGH (aCGH), Lenz et al. detected gains or amplifications of 2p in 35% of GCB- and 12% of ABC-DLBCL. Restricting the analysis to amplifications, the authors detected copy number increases of larger than 3.5 in 28% and 5% of all samples for the GCB and ABC subgroup, respectively [100]. While all three analyses of largely overlapping lymphoma samples agree that gain of *REL* is a frequent genomic alteration in DLBCL, there is a discrepancy between the reported subgroup frequency. Nevertheless, two of the three studies detected a clear bias towards *REL* amplification in GCB-DLBCL.

In agreement with a higher abundance of *REL* overrepresentation in the GCB-DLBCL subgroup, Feuerhake et al. found 17% (10/57) and 5% (1/22) *REL* amplifications in GCB- and ABC-DLBCL-classified samples, respectively [101]. In another published patient group, 28% of GCB (5/18), 17% of ABC (2/12) and 15% of type 3 DLBCL (2/13) display *REL* amplifications with at least four copies [77], hence further corroborating this tendency. Similarly, an additional study observed an association of 2p15-p16.1 gain predominantly with the GCB-DLBCL subtype with gains in 33% (6/18) of GCB-DLBCL patients whereas gains were detected in only 7% (2/28) of ABC-DLBCL patients [102]. In a cohort of 114 DLBCL cases, Jardin et al. observed *REL* gains in 18% (21/114) of all samples with a distribution amongst the GCB and ABC cluster of 6/64 (9%) and 10/27 (37%), respectively, while the remaining 5/23 (22%) samples were not classified. Bea et al. did not distinguish subtypes by gene expression profile and found 5/64 gains and 4/64 amplifications summing up to 14% (9/64) of DLBCL patients with 2p14-p16 overrepresentation [103].

In summary, gains or amplifications in 2p16.1 containing the *REL* locus are highly abundant in DLBCL with an overall markedly higher prevalence in the GCB-DLBCL compared to the ABC-DLBCL subset. As NF-κB target genes are not enriched in the subgroup of GCB-DLBCL it was suggested that this could point to functional importance of *REL* amplification early during the process of malignant transformation. Lymphoma cells could then locate to another environment, where an absence of signals activating c-Rel transcriptional activity renders the presence of *REL* gain irrelevant [74]. *REL* gain would essentially act as a “hit-and-run” oncogenic event, which could explain the absence of a clear clinical correlation.

### 4.2. REL Gains in Follicular Lymphoma and Transformed Follicular Lymphoma

Follicular lymphoma (FL) is a GC B cell-derived lymphoma characterized by an indolent, though highly variable, clinical course, reflected by large numbers of heterogeneous lymphoma-associated molecular aberrations. The hallmark of FL is the t(14;18)(q32;q21) translocation, which places expression of the anti-apoptotic Bcl-2 protein under control of the immunoglobulin heavy chain locus, leading to significant overexpression [104,105]. Many healthy individuals contain B cells carrying *Bcl2* translocations, and the frequency increases with age [106]. It is likely that *Bcl2* overexpression in the germinal center increases the risk of additional mutations that ultimately result in the development of FL. Around 45% of FL transform into the more aggressive B-NHL subtype of DLBCL, referred to as transformed FL (tFL). Amongst the additional molecular characteristics frequently detected in FL and paired tFL biopsies are gains of the *REL* locus.

In a small study Nagy et al. report a gain at 2p14-pter in one out of five patients of FL transformed to DLBCL, while no gain in this respective locus was found for the corresponding FL sample [107]. Hough et al. compared not only FL and tFL, but also included de novo DLBCL in their analysis of genomic aberrations. Additionally, this group did not detect any gains at 2p12-16 in FL (0/18), but in 17% (4/23) of tFL and in 33% (6/18) of de novo DLBCL [108].

Goff et al. report amplifications at 2p13-16 in one of six (17%) FL and in half (50%, 3/6) of the respective tFL using CGH. Interestingly, two patients of this study who presented with amplifications at the tFL stage showed low level copy number changes of *REL* detected by quantitative PCR already at the FL stage, despite the absence of 2p13-16 imbalances in the CGH analysis [109]. In another study, including 10 patients with paired FL and tFL biopsies, 10% (1/10) of FL and 20% (2/10) of tFL showed 2p16 amplifications by aCGH analysis [110]. Using quantitative PCR Davies et al. found *REL* locus amplifications in two out of 20 tFL patients of which one case presented with the amplification already before transformation [111].

In a study aiming to elucidate clonal evolution during transformation of FL, Pasqualucci et al. used whole exome sequencing (WES) and SNP array analysis to compile genomic profiles of FL and tFL patient samples. In their discovery panel of 12 matched FL/tFL pairs, the authors report gains/amplifications of genetic regions including the *REL* locus in 33% (4/12) of FL and 50% (6/12) in the respective tFL patients. In a screening panel of 39 tFL patient samples, high copy number amplifications of 2p16.1 were detected in 31% (12/39) of cases [112]. Kwiecinska et al. found a gain at 2p15-16.1 in 20% (3/15) of FL patients and 41% (12/29) of tFL patients using aCGH. In addition, this study includes samples of de novo diagnosed DLBCL patients for which only one of 29 samples was positive for a 2p15-16.1 gain. These data are supported by gene copy number assessment by qPCR performed in a subset of this panel with gains in *REL* in one of seven FL (14%), 39% (7/18) tFL and one of 25 de novo DLBCL [113].

In the study with the largest cohort of FL/tFL patients included in the present overview Bouska et al. used single nucleotide polymorphism array to define recurring copy number abnormalities (rCNA) [114]. Considering any copy number gains on a chromosomal level affecting the *REL* locus, the authors report a copy number increase in 24% (48/198) of FL and 30% (24/79) of tFL. When including only amplifications still 10% (19/198) of FL and 13% (10/79) of tFL samples show these genetic aberrations. The method also allowed the authors to analyze copy number changes of shorter DNA segments covering only 13 genes (rCNA693) or eight genes (rCNA1030), both of which include the *REL* locus and cover either gains and amplifications or only amplifications, respectively. In these smaller gained fragments, an important role for c-Rel is more likely than in larger fragments containing many more genes. Considering both gains and amplifications (rCNA693) this class of rCNA showed copy number increases in 11% (21/198) of FL and 19% (15/79) of tFL. Interestingly, even limiting the analysis to only amplifications (rCNA1030) still resulted in 9% (17/198) of FL and 11% (9/79) of tFL cases presenting with amplifications of relatively short DNA fragments including the *REL* locus [114] (A. Bouska and W.-C. Chan, personal communication).

To conclude, copy number increases of gene segments including the *REL* gene are detected in FL. Nevertheless, *REL* gains and amplifications are an even more frequent characteristic at the transformed stage of this human B cell lymphoma subtype (tFL). Interestingly, the tFL gene expression phenotype resembles GCB-DLBCL [111], which has a higher prevalence of *REL* gains than the ABC-DLBCL subtype as highlighted above.

### 4.3. REL Gains in Primary Mediastinal B Cell Lymphoma

PMBCL originates in the mediastinum, probably with the involvement of the thymus [115] and thymic medullary B cells are the proposed cells of origin [68,116]. Originally grouped within the DLBCL lymphoma entity, PMBCL was recognized as a distinguishable separate lymphoma type later on [115]. Subsequent studies using gene expression profiling [117,118] suggested that PMBCL shares characteristics with cHL on the molecular phenotype level [117,118].

Two of the publications cited above for DLBCL also assessed 2p overrepresentations in PMBCL samples. While Bea et al. detected gains in 47% of analyzed samples, Lenz et al. report gains including amplifications for 26% and amplifications for 19% of all PMBCL cases [99,100]. In an independent cohort, a gain in 2p was detected in 27% (7/26) PMBCL biopsies. Two of these cases display high-level *REL* amplifications of 5- and 10-fold as quantified by Southern blot [119]. Moreover, Southern blot quantification for a small number of 11 PMBCL patients showed copy numbers ≥4 in 36% (4/11) of samples [120]. Using CGH another study detected an overrepresentation of 2p14-16 in 19% (8/43) of PMBCL cases [121]. Interphase cytogenetics employing fluorescence in situ hybridization (FISH) analysis of material from 20 of these patients substantiated two gains and three amplifications previously found by CGH in this selected sample set and revealed in addition eight gains and two amplifications, which amounts to a total number of 15 *REL* copy number changes in 20 samples [122].

### 4.4. REL Gains in Classical Hodgkin Lymphoma

cHL accounts for about 10% of human mature B cell lymphomas [68]. A hallmark of cHL are mononucleated Hodgkin cells and multinucleated Reed-Sternberg cells. Remarkably, these Hodgkin and Reed-Sternberg (HRS) cells constitute only 1% of tumor cells. Although HRS cells have lost major B cell gene expression characteristics and even acquired markers of other lineages, they originate from GC B cells based on their rearranged and somatically mutated Ig gene loci [123,124].

In a study of 41 cHL tumors, Joos et al. detected gains in 2p by CGH as the most frequent alteration in 54% of cases [125]. Interphase cytogenetic analysis of 17 samples from the same cohort by FISH demonstrates five gains and three amplifications. Only in one case, the amplification contradicted the CGH data summing up to an overrepresentation of the *REL* locus in 41% (7/17) of cHL patients substantiated by both methods [36]. A similar assessment of interphase cytogenetic by FISH of 31 cHL biopsies showed *REL* locus gains in 35% (11/31) including eight cases with high-level amplifications (26% of total cases) [83]. Applying aCGH profiling on 53 cHL specimens Steidl et al. detected gains in the 2p15-16.1 genomic locus in 28% of patients [126]. In a more recent publication, whole genome sequencing determined significant copy number gains ≥4 of the chromosomal segment containing the *REL* locus in 40% (8/20) of cHL patients [127]. Given the very strong NF-κB activation induced by Epstein Barr Virus (EBV)-encoded proteins, it would be interesting to assess the correlation between *REL* gain and EBV status in HL. However, to our knowledge to date, this has not been clarified. Finally, the 2p16.1 *REL* locus was identified as a susceptibility locus for cHL in a genome-wide association study (GWAS) through association to a single nucleotide polymorphism rs1432295 [85]. The association was confirmed in a study with over 1400 HL cases [86]. These studies provide strong evidence for a role for c-Rel gain in the pathogenesis of cHL.

## 5. *REL* Gain in Relation to c-Rel Protein Abundance and Localization

As outlined above, a gain of a chromosomal area including the *REL* genomic locus is a highly frequent event in GC-derived human B cell lymphomas. However, the functional consequences of these recurrent gains remain elusive. In order to gain first insights, several studies assessed the relation of *REL* genomic status and c-Rel expression level at the mRNA and/or protein level as well as its subcellular localization. As a transcription factor, one would expect that increased function should be reflected by higher amounts of nuclear c-Rel.

### 5.1. Expression and Localization of c-Rel in DLBCL

In a large cohort of 224 DLBCL patients, *REL* mRNA expression assessed by microarray correlated with amplifications (CGH signal ratios > 1.5) and gains (CGH signal ratios > 1.25) at 2p14-16 [99]. *REL* mRNA is significantly higher in GCB-DLBCL samples with 2p14-p16 amplifications (n = 8) and gains (n = 7) [101] compared to samples with normal 2p status (n = 72). In ABC-DLBCL samples a similar trend was observed, which did not reach statistical significance [99]. Similarly, two other studies observed augmented *REL* mRNA levels in specimens with *REL* locus gains in DLBCL patient cohorts of 127 [101] and 114 [128] subjects, respectively. Together, these studies indicate that gain at 2p14-16 is associated with increased c-Rel mRNA levels in DLBCL.

Other publications focused on the assessment of c-Rel nuclear protein levels. Houldsworth et al. observed heterogeneity with regard to c-Rel nuclear accumulation based on immunofluorescence and did not detect a correlation of nuclear c-Rel with *REL* amplification. At least 70% positive c-Rel nuclei were found in 44% (17/39) of cases with a higher prevalence in the ABC-DLBCL subgroup despite the higher percentage of *REL* amplifications within the GCB-DLBCL subgroup of this cohort [77]. Another study, defining positive nuclear presence when 30% or more of tumor cells exhibited nuclear staining, detected c-Rel nuclear presence in 64% of 113 DLBCL samples. In this work, c-Rel was found to be the NF-κB subunit with the highest clearly detectable nuclear presence when comparing the five NF-κB family members. In this cohort, detectable nuclear presence of c-Rel was equally distributed amongst the GCB- and ABC-DLBCL subgroups and correlated with favorable prognosis [129]. Curry et al. set a cutoff for positivity of c-Rel nuclear protein in 20% of neoplastic cells, which results in a comparable frequency of 65% (44/68) of DLBCL cases that fall into this category [97]. The authors did not observe a significant correlation of nuclear c-Rel with the DLBCL subgroups or overall survival of patients. However, this data set suggests a trend for worse survival of patients with positive nuclear c-Rel expression within the GCB-DLBCL cluster [97] and therefore does not support the correlation with a better prognosis observed by Odqvist et al. [129]. With a clearly lower cutoff defining a sample as positive for the nuclear presence of c-Rel when 5% of tumor cells showed nuclear c-Rel staining, Li et al. found only 26% (137/460) of DLBCL patients with positive nuclear presence of c-Rel and a similar distribution of cases amongst the subclasses. *REL* mRNA is significantly higher in the GCB- compared to the ABC-DLBCL group, but *REL* mRNA levels and the nuclear presence of c-Rel showed no correlation in this large cohort [130]. Rodig et al. found nuclear c-Rel staining in 18% (28/160) of DLBCL samples that were not further subclassified [131]. Pham et al. used an ELISA-based approach to quantify binding of p52, RelA, c-Rel, and RelB to a consensus NF-κB site in 19 DLBCL-derived cell lines (14 GCB- and 5 ABC-DLBCL) as well as in nine GCB- and five ABC-DLBCL patient samples. In both cell lines and patient-derived material, RelA NF-κB site-binding was over two-fold higher in ABC-DLBCL samples, whereas c-Rel NF-κB site-binding was over two-fold higher in GCB-DLBCL samples [132]. Overall, in spite of increased *REL* mRNA levels, no clear correlation between genomic gain at 2p14-16 and increased nuclear presence of c-Rel could be detected by immunohistochemistry. However, immunohistochemistry also did not reveal a clear difference in nuclear presence between GCB- and ABC-DLBCL cases, while an assay based on DNA-protein interactions indicated elevated c-Rel DNA binding in GCB-DLBCL. This discrepancy indicates that the relationship between 2p14-16 gain and c-Rel nuclear presence and function should be investigated by additional means before definitive conclusions can be reached.

### 5.2. Localization of c-Rel in PMBCL

In order to study the relation of genomic *REL* gains and c-Rel protein level on a single cell basis, Weniger et al. combined FISH interphase cytogenetics with immunofluorescence staining (FICTION, fluorescence immunophenotyping, and interphase cytogenetics as a tool for investigation of neoplasms). This analysis demonstrated a positive correlation of *REL* genomic overrepresentation, and c-Rel nuclear staining for the 10 analyzed PMBCL samples [122]. In two other publications, c-Rel nuclear localization was observed by immunohistochemistry in five of six PMBCL patient samples investigated by Savage et al. [118] and in all of seven PMBCL biopsies tested in the other study [101]. In a considerable cohort of 48 PMBCL patients, 65% (31/48) of samples were characterized by the presence of nuclear c-Rel [131].

### 5.3. Localization of c-Rel in cHL

In a cohort of 25 cHL patients, Barth et al. demonstrated a strong correlation of genomic gains at 2p14-16 with nuclear c-Rel staining in HRS cells. Of the 12 cases with *REL* copy number gains, seven showed positive nuclear c-Rel staining in at least 70% of HRS and five in more than 30% of HRS cells. On the other hand, the majority of samples with unaltered *REL* genomic status displayed less than 30% of positive HRS cells [36]. In addition, nuclear c-Rel was detected in two independent studies in 80% (23/25) [133] and even 86% (51/59) [134] of cHL patient material.

In summary, the publications cited above provide strong evidence for frequent nuclear presence of c-Rel in PMBCL and cHL. Furthermore, in both entities, one study provides evidence for a positive correlation of *REL* gene locus amplification and c-Rel expression level accompanied by frequent observations of nuclear c-Rel protein accumulation. In contrast, in DLBCL the picture is more complex: Overall, it seems that gain of 2p14-16 is associated with higher *REL* mRNA levels, fitting with the more frequent occurrence of gains in GCB-DLBCL, which overall has higher *REL* mRNA levels compared to ABC-DLBCL. However, the literature does not provide evidence for a correlation between nuclear presence of c-Rel and gain of 2p14-16 or DLBCL subtype. This could be due to many factors, including technical limitations as well as the overall heterogeneity of DLBCL. As indicated above, it is noteworthy that gene expression analyses demonstrate that PMBCL is related to cHL with closer resemblance than to other DLBCL subtypes [117,118]. Interestingly, shared features of cHL and PMBCL are an association with high NF-κB activity as well as lack of detectable BCR expression [118,122,123]. It is also worth mentioning that cell line-based studies suggest that predominant nuclear or cytoplasmic c-Rel localization does not necessarily constitute a prognostic clue for malignant transformation [135]. Finally, c-Rel localization and expression level could be characteristic and specific for the lymphoma cell of origin, as mentioned by Gilmore and Gerondakis [9].

## 6. *BCL11A* Is Frequently Co-Amplified with *REL*

Studies that did not find a positive correlation between *REL* gene amplification and c-Rel protein expression questioned whether *REL* is indeed the target of the 2p16.1 locus gains and amplifications. In fact, *REL* is not the only gene in the commonly amplified genomic region. Amongst others, the *BCL11A* gene also maps to 2p16.1 and is located only within 300 kB of *REL* (according to genomic location of BCL11A ENSG00000119866 accessible on www.ensembl.org (human GRCh38.p5)). The Krüppel zinc finger transcription factor Bcl-11a is essential for lymphocyte development and in particular B cells are virtually absent in Bcl11a-deficient mice [136]. Intriguingly, Bcl-11a is highly expressed in the GC, and a translocation involving *BCL11A* and the Ig heavy chain locus has been discovered in B cell malignancies [137].

Several studies provide evidence for a frequent co-amplification of the *REL* and *BCL11A* loci in the B cell lymphoma subtypes discussed above. In four of seven DLBCL cases analyzed by Fukuhara et al. using aCGH both *REL* and *BCL11A* are gained, whereas three cases show an exclusive *REL* gain and these genomic gains correlate with mRNA expression levels [138]. In a study by Bea et al., all nine DLBCL patients with 2p14-p16 gains or amplifications show elevated copy numbers for both *REL* and *BCL11A* assessed by real-time quantitative polymerase chain reaction (RQ-PCR) [103]. Similarly, in a large cohort of 224 patients, the vast majority of cases of GCB- and ABC-DLBCL as well as PMBCL show concomitant copy number changes of *REL* and *BCL11A* according to RQ-PCR [99]. Moreover, in a series of 15 PMBCL cases, copy numbers for *BCL11A* and *REL* were shown to be simultaneously increased [122,139]. Weniger et al. observed nuclear Bcl-11a protein in 88% (14/16) of analyzed PMBCL. However, only in 25% (4/16) of cases, *BCL11A* copy number alterations, transcript levels and nuclear protein clearly correlate [139].

In FL and its transformed counterpart (tFL) both *REL* and *BCL11A* are affected by copy number gains. Kwiecinska et al. detect higher copy numbers of *REL* in one of seven (14%) FL and 7/18 (39%) tFL cases. With the exception of one case, the same patients also show concomitant gains of *BCL11A* (1/7 FL (14%), 6/18 tFL (33%)) [113]. Similarly, two further studies report gains and amplifications in FL and tFL that include both the *REL* and the *BCL11A* gene locus [112,114].

Lastly, more than 90% (10/11) of cHL cases with *REL* amplification and gains show concomitant *BCL11A* amplification and gains, respectively. In two cases (2/21) of this cohort with balanced *BCL11A* status, the authors found indications for modifications of the *REL* locus without affecting *BCL11A* [83].

Together, it emerges that in the majority of analyzed lymphoma cases the *BCL11A* gene is co-amplified with the *REL* gene. Genetic loss of study functions in knockout mice strongly implicate both genes in B cell differentiation and function, indicating that both could also have a role in B cell malignancies. However, the close genomic proximity leaves the possibility that one of these genes could be a passenger in the gained genomic material selected by the dominant effects of the driver gene. Hence, both c-Rel and Bcl-11a represent interesting candidates for prospective gain-of-function studies, in particular with regard to the investigation of compound effects.

## 7. Investigating the Oncogenic Role of c-Rel in Mouse Models

Interestingly, c-Rel locus amplification was also detected in a recent study where B cell lymphomas were induced through random insertion of inactivating transposons in the context of Bloom-deficiency in order to enhance loss of heterozygosity. Over 60% of the murine lymphomas analyzed for copy number variations (10/16 samples) harbor chromosomal gains on chromosomal region 11qA1-B1.3, which corresponds to the human region on chromosome 2p15-16. The minimal overlap region contained 27 genes, amongst them *Rel* and *Bcl11a* [140]. This result implicates gain of *Rel* also in murine lymphomagenesis. A role for c-Rel could further be supported by transposon integrations in the murine *Rel* locus detected in this screen. However, whether the insertions enhance or impair c-Rel expression remained unclear. The employed transposons were designed to induce transcriptional termination in both possible integration orientations. Eleven of sixteen integrations occurred in intron 5, all in the same orientation, which could lead to production of a truncated N-terminal c-Rel. As this truncated protein would contain the DNA binding domain but lack the transactivation domains, its existence would not support an oncogenic role for enhanced c-Rel activity. On the other hand, one can speculate that insertions increase *Rel* transcription through cryptic promoter elements located in the transposon inverted terminal repeats. Furthermore, four transposons integrated into one orientation in the *Rel* 3′ untranslated region [140], which might relieve c-Rel expression from translational repression mechanisms.

In spite of the pervasive evidence for an oncogenic role of c-Rel in lymphoma functional analyses investigating the elusive roles of *REL* gene locus amplification and aberrant splicing are missing to date, mostly due to the lack of suitable in vivo models. A general oncogenic role for c-Rel in lymphoma was put into doubt by a recent publication reporting that loss of c-Rel through gene knockout correlated with earlier disease onset in the Eμ-Myc and pEμ-B29-TCL1 lymphoma/leukemia mouse models. The authors suggest in the case of Eμ-Myc-driven lymphomagenesis that the results could be explained by the observed downregulation of the tumor suppressor Bach2 in Eμ-Myc c-Rel-deficient mice [141]. However, although the Eμ-Myc lymphoma mouse model has provided invaluable insight into general aspects of lymphoid transformation, it represents mainly pre-B cell tumors [142], which might not adequately reflect the role of c-Rel in human cHL and B-NHL disease entities. Furthermore, as the c-Rel deficiency was not limited to B cells in these studies, B cell-extrinsic factors could also contribute to the accelerated lymphomagenesis in c-Rel-deficient mice. As c-Rel-deficiency diminishes regulatory T cell exerted control over anti-tumor effector T cell responses, while leaving the effector T cell responses themselves largely unaffected [143], these factors reside mostly likely outside the T cell lineage.

## 8. c-Rel as a Therapeutic Target

Given the manifold evidence for an involvement of c-Rel in human diseases, the concept of c-Rel as a therapeutic target has been pursued. As outlined above, GC-derived lymphomas could be candidates for c-Rel inhibition, probably in combination with other therapeutic agents. However, c-Rel is also implicated in solid tumors, for example, through aberrant activation by mutant K-ras in solid malignancies [144]. In addition to its effects on tumor cells, c-Rel inhibition might have the additional benefit of enhancing anti-tumor immunity through inhibiting the function of regulatory T cells [143]. In this study, the authors provide evidence that the xanthine derivative pentoxifylline (PTXF) causes selective degradation of c-Rel and impacts regulatory T cells comparable to genetic c-Rel ablation. In this context, PTXF treatment can delay tumor growth and shows an additive effect in combination with checkpoint blockade [143]. In addition, treatment with siRNA targeting c-Rel delivered in micelles via intraperitoneal (i.p.) injection in a mouse model of imiquimod (IMQ)-induced psoriasis was shown to prevent or mitigate disease symptoms [145].

Specifically targeting c-Rel could have advantages over general NF-κB inhibition, which acts systemically and has dramatic effects on non-immune cells [146,147]. The viability of c-Rel knockout mice and their defects primarily affecting the immune system corroborate this strategy. In vitro knockdown of c-Rel in human HL cell lines [148] as well as in mouse WEHI-231 lymphoma cells [149] impaired cellular expansion, thus providing evidence for a therapeutic effect of c-Rel inhibition in lymphoma. In light of the fact that transcription factors are not regarded as easily targetable [147], it is especially important to thoroughly elucidate the specific signaling pathways that lead to c-Rel activation. For example, the peptidyl-prolyl isomerase Pin-1 was shown to regulate c-Rel nuclear translocation [64]. Inhibition of Pin-1 through Juglone (5-hydroxy-1,4-naphthoquinone), an irreversible inhibitor of parvulin peptidyl-prolyl isomerases as well as Pin-1 knockdown interfered with the expansion of PMBCL and HL cell lines [64]. Notwithstanding the conceptual difficulties, compounds directly interfering with c-Rel were reported to show some activity: The NF-κB inhibitor dehydroxymethylepoxyquinomicin (DHMEQ) was reported to target conserved cysteine residues and to form adducts with c-Rel as well as other NF-κB subunits, thus impairing their DNA-binding ability [150,151]. However, the biological consequences of this impaired binding remain unclear. The inhibitor CM101 targeting at least c-Rel and RelA induces proliferation arrest and apoptosis in human B cell lymphoma cell lines. Interestingly, in particular, cell lines expressing high levels of c-Rel were sensitive towards CM101 [152]. In a small molecule screen based on a biochemical assay determining c-Rel DNA binding, Shono et al. identified compounds that inhibit NF-κB and are claimed to show specificity for c-Rel in particular. Subsequently, derivatives of these compounds, namely the thiohydantoin IT-603 and the naphthalenethiobarbiturate IT-901, were employed in preclinical mouse models: The effects included amelioration of graft-versus-host disease (GVHD) without affecting antitumor responses and slowing the expansion of xeno-transplanted human B cell lymphoma cell lines [153,154]. However, another study treating chronic lymphocytic leukemia cells with IT-901 suggest inhibitory activity of this compound also on RelA [155]. Further studies are required to assess the efficacy of c-Rel inhibition for human lymphoma through both direct effects on the tumor cells and indirect effects on its immune microenvironment. Such studies should include CRISPR-based knockouts of c-Rel in lymphoma cell lines to validate the effects of shRNA-mediated c-Rel knockdown as well as of inhibitor treatments, which are both prone to off-target effects. Furthermore, the effects of c-Rel inactivation on lymphoma and its microenvironment should be tested in immunocompetent animal models. It is possible that c-Rel inhibition will reveal its potential mostly in combination with other therapeutic agents. An interesting question is also whether lymphomas with c-Rel gain are particularly susceptible to c-Rel inhibition or whether they display other specific vulnerabilities due to direct of indirect consequences of c-Rel gain that could be exploited therapeutically.

## 9. Conclusions

The studies discussed in this review highlight the remarkably frequent gains and amplifications of the human gene locus 2p16.1 containing the *REL* gene in several human B cell lymphoma subtypes. FL, DLBCL, and PMBCL as well as cHL are characterized by highly frequent occurrence of this particular genetic aberration, the latter two lymphoma subtypes even more prominently. It is noteworthy that the *REL* gene locus is not only implicated in human B cell lymphoma, but also in autoimmunity. In fact, genome-wide association studies reported associations of single nucleotide polymorphisms (SNPs) within the *REL* gene locus for human diseases with immunological etiology, including rheumatoid arthritis [156,157], psoriasis [158], ulcerative colitis [159] and celiac disease [160,161]. Interestingly, not only are most human B cell lymphomas of GC or post-GC origin, but deregulated GC reactions are also implicated in the development of autoimmunity [162]. Furthermore, patients with certain immune diseases have an increased risk to develop lymphoma later in life [106,163,164]. Interestingly, a single nucleotide polymorphism near the *REL* gene locus was also linked to cHL [85,86]. This SNP is linked to an enhancer, and it is also associated with rheumatoid arthritis and psoriatic arthritis, potentially pointing to common disease mechanisms supported by alterations in c-Rel regulation.

In conclusion, through the work of multiple groups over many years a strong case has been made for an association of genetic alterations affecting the *REL* gene locus both with human lymphoma and autoimmunity. The intriguing question how gain of c-Rel function contributes to the pathogenesis of these human diseases on a molecular level remains to be comprehensively addressed as well as the question whether c-Rel gain constitutes or causes a vulnerability that can be exploited therapeutically. Addressing these questions on a functional level will require the development of in vivo models for c-Rel gain-of-function, as pointed out more than a decade ago [38]. Of highest interest in this context are studies elucidating consequences of enhanced c-Rel signaling in late-stage B cell differentiation—representing the cell types that are the malignant cell type or are major disease drivers or contributors in human lymphoma and autoimmunity, respectively.

## Figures and Tables

**Table 1:** Frequency of *REL* gene locus gains in human B cell lymphoma subtypes. The frequencies of gains including amplifications are displayed. In parenthesis, the fraction of the total number of investigated patients is given. The average percentage of *REL* gain per subtype (bold, last row) was calculated using the sum of total reported patient numbers. The average for DLBCL includes data from both GCB- and ABC-DLBCL subgroups. The table summarizes the literature with no claim to completeness of published datasets. *^1^ Data were confirmed by qPCR. *^2^ Numbers for two analyzed patient panels (discovery panel, screening panel) are given. *^3^ Numbers refer to aCGH analysis. Part of the patient panel was additionally analyzed by qPCR. *^4^ largely redundant cohorts. Numbers displayed as given in Houldsworth et al. [89]. *^5^ 23 samples of this DLBCL cohort were unclassified. *^6^ apparently based to large extent on the same cohort. *^7^ Weniger et al. [122] selected five samples with and 15 samples without gain/amplification from the Bentz at al. [121] cohort. *^8^ Barth et al. [36] studied samples from the Joos et al. [125] cohort. Cases consistently gained in both CGH and FICTION analysis were considered positive. Method abbreviations: aCGH, array comparative genomic hybridization; CGH, comparative genomic hybridization; FICTION, FISH and combined immunophenotyping and interphase cytogenetics; FISH, Fluorescence in situ hybridization; QMPSF, quantitative multiplex PCR of short fragments; qPCR, quantitative polymerase chain reaction; SB, Southern Blot; SNP array, single nucleotide polymorphism array; WES, whole exome sequencing; WGS, whole genome sequencing.

Reference	Method	FL	tFL	DLBCL	GCB-DLBCL	ABC-DLBCL	PMBCL	cHL
Nagy et al. [107]	CGH	0% (0/5)	20% (1/5)					
Hough et al. [108]	CGH	0% (0/18)	17% (4/23)	33% (6/18)				
Goff et al. [109] *^1^	CGH, qPCR	17% (1/6)	50% (3/6)					
Martinez-Climent et al. [110]	aCGH	10% (1/10)	20% (2/10)					
Davies et al. [111]	qPCR	5% (1/20)	10% (2/20)					
Pasqualucci et al. [112] ^*2^	WES, SNP array	33% (4/12)	50% (6/12) 31% (12/39)					
Kwiecinska et al. [113] *^3^	aCGH, qPCR	20% (3/15)	41% (12/29)	3% (1/29)				
Bouska et al. [114]	SNP array	24% (48/198)	30% (24/79)					
Houldsworth et al. [89] Rao et al. [90]	SB			23% (26/111) *^4^				
Bea et al. [103]	CGH			14% (9/64)				
Jardin et al. [128]	QMPSF			22% (5/23) *^5^	37% (10/27)	9% (6/64)		
Rosenwald et al. [94] *^6^	qPCR				15% (17/115)	0% (0/73)		
Bea et al. [99] *^6^	CGH				17% (15/87)	15% (12/77)	47% (9/19)	
Lenz et al. [100] *^6^	aCGH				35% (72)	12% (74)	26% (31)	
Houldsworth et al. [77]	SB				28% (5/18)	17% (2/12)		
Feuerhake et al. [101]	qPCR				17% (10/57)	5% (1/22)	3% (1/34)	
Tagawa et al. [102]	aCGH				33% (6/18)	7% (2/28)		
Joos et al. [119]	CGH						27% (7/26)	
Bentz et al. [121] *^7^	CGH						19% (8/43)	
Weniger et al. [122] *^7^	FISH						75% (15/20)	
Palanisamy et al. [120]	SB						36% (4/11)	
Martin-Subero et al. [83]	FICTION							35% (11/31)
Joos et al. [125] *^8^	CGH							54% (22/41)
Barth et al. [36] *^8^	CGH, FICTION							41% (7/17)
Steidl et al. [126]	aCGH							28% (53)
Salipante et al. [127]	WGS							40% (8/20)
Average		20%	30%	17%	21%	9%	28%	39%

**Table 2:** Frequency of *REL* gene locus amplifications in human B cell lymphoma subtypes. The frequencies of amplifications are displayed. Studies summarized in Table 1 were included in this table if amplifications were defined as a *REL* copy number of ≥4 copies or *REL* gene ratio of ≥2. In parenthesis, the fraction of the total number of investigated patients is given. The average percentage of *REL* amplification per subtype (bold, last row) was calculated using the sum of total reported patient numbers, the average for DLBCL includes data from both GCB- and ABC-DLBCL subgroups. *^1^ aCGH results distinguishing gains and amplifications only provided for 15 paired FL/tFL samples. Part of the total patient panel was additionally analyzed by qPCR. *^2^ Jardin et al. [128] did not distinguish gains/amplifications but provided gene ratios for all samples. Numbers presented here are based on a *REL* gene ratio of ≥2 representing amplifications. For additional information on studies and provided numbers as well as method abbreviations see legend of Table 1.

Reference	Method	FL	tFL	DLBCL	GCB-DLBCL	ABC-DLBCL	PMBCL	cHL
Martinez-Climent et al. [110]	aCGH	0% (0/10)	20% (2/10)					
Pasqualucci et al. [112]	WES, SNP array	8% (1/12)	8% (1/12) 31% (12/39)					
Kwiecinska et al. [113] *^1^	aCGH, qPCR	7% (1/15)	20% (3/15)					
Bouska et al. [114]	SNP array	10% (19/198)	13% (10/79)					
Houldsworth et al. [89] Rao et al. [90]	SB			23% (26/111)				
Jardin et al. [128] *^2^	QMPSF			4% (1/23)	7% (2/27)	2% (1/64)		
Rosenwald et al. [94]	qPCR				15% (17/115)	0% (0/73)		
Houldsworth et al. [77]	SB				28% (5/18)	17% (2/12)		
Feuerhake et al. [101]	qPCR				17% (10/57)	5% (1/22)	3% (1/34)	
Joos et al. [119]	CGH						8% (2/26)	
Bentz et al. [121]	CGH						0% (0/43)	
Weniger et al. [122]	FISH						25% (5/20)	
Palanisamy et al. [120]	SB						36% (4/11)	
Martin-Subero et al. [83]	FICTION							26% (8/31)
Joos et al. [125]	CGH							2% (1/41)
Barth et al. [36]	CGH, FICTION							12% (2/17)
Salipante et al. [127]	WGS							30% (6/20)
Average		9%	18%	12%	16%	2%	9%	16%
